# Can a Theater Acting Intervention Enhance Inhibitory Control in Older Adults? A Brain-Behavior Investigation

**DOI:** 10.3389/fnhum.2021.583220

**Published:** 2021-03-18

**Authors:** Aishwarya Rajesh, Tony Noice, Helga Noice, Andrew Jahn, Ana M. Daugherty, Wendy Heller, Arthur F. Kramer

**Affiliations:** ^1^Beckman Institute, University of Illinois at Urbana-Champaign, Champaign, IL, United States; ^2^Department of Psychology, University of Illinois at Urbana-Champaign, Champaign, IL, United States; ^3^Elmhurst College, Elmhurst, IL, United States; ^4^fMRI Laboratory, University of Michigan, Ann Arbor, MI, United States; ^5^Department of Psychology, Wayne State University, Detroit, MI, United States; ^6^Department of Psychology, Northeastern University, Boston, MA, United States

**Keywords:** acting, AXCPT, cognitive intervention, proactive control, reactive control

## Abstract

**Purpose:** Studies of reactive and proactive modes of inhibitory control tend to show age-related declines and are accompanied by abnormalities in the prefrontal cortex. We explored which mode of inhibitory control would be more amenable to change and accrue greater benefits following engagement in a 4-week theater acting intervention in older adults. These gains were evaluated by performance on the AX-CPT task. We hypothesized that an increase in proactive control would relate to an increase in AY errors and a decrease in BX errors. In contrast, an increase in reactive control would be associated with a decrease in AY errors, no change in AY reaction time, and an increase in BX response time. Further, we posited that an increase in behavioral proactive control would accompany greater cue versus probe activity for previously identified regions in the prefrontal cortex. In contrast, an increase in behavioral reactive control would be accompanied by greater probe activation in these identified brain areas.

**Materials and Methods:** The participants were 179 community-dwelling adults aged 60–89 years who were on average, college-educated. Participants were pseudo-randomly assigned to either an active-experiencing acting intervention condition (*n* = 93) or the active control condition (*n* = 86); participant assignment was subject to time of enrollment. Participants in both groups were trained by theater-actor researchers with expertise in acting interventions. In contrast to the active control participants who attended a course on theater acting, the acting-intervention group was required to consistently deploy proactive and reactive control mechanisms. Both groups met two times/week for 75-min for 4 weeks. Participant brain-behavioral performance on the AX-CPT task was evaluated prior to and after this four-week period.

**Results:** No intervention effects were found in favor of proactive control. Behavioral evidence in favor of reactive control was weak. Brain-related benefits to reactive control was illustrated by greater probe-activation in Brodmann areas 6 and 8, relative to controls and pre-intervention.

**Conclusion:** We found some evidence for improvements in reactive control via brain measures, attributed to engagement in the acting intervention.

## Introduction

Aging is associated with multiple changes in brain structures and functions that, in greater severity, constitute pathology, such as Alzheimer’s disease. Yet, age-related changes across brain regions and functions are not uniform. For example, cognitive control —which reflects the ability to manage disruptive effects of irrelevant information on the active maintenance of task goals— declines with aging, accompanied by abnormalities in the prefrontal cortex (Braver et al., 2001; Craik and Bialystok, 2006; Bailey et al., 2008; Burgess et al., 2011; Bugg, 2014; Banducci et al., 2017). The dysfunction of cognitive control is associated with disruptions in working memory, fluid intelligence, and adaptive reasoning (Gottfredson, 1997; Unsworth, 2010; Unsworth et al., 2014), which are predictive of behavioral outcomes. Thus, identifying the pattern and sources of individual differences in cognitive control in older adults may help identify potential avenues to promote maintenance into older age. This study explores age-related differences in inhibitory aspects of cognitive control (Braver, 2012) attributed to a theater-acting-training intervention. We use “inhibitory control” as a useful organizing term to characterize these aspects of cognitive control. To this end, both behavioral and neurofunctional changes in inhibitory control are assessed using the AX-CPT task paradigm developed by Braver et al. (2009).

Recent studies indicate that inhibitory control is composed of two distinct forms of inhibition - proactive and reactive control (Braver, 2012). Proactive control requires the early recruitment of goal-relevant information in preparation of a cognitively demanding event and attentional monitoring of that information until the event goal is met. This inhibition mechanism attempts to ensure that the salience of interfering information is minimized to the extent possible. In contrast, reactive control is transiently activated to resolve interference after an event has occurred and conflict with the event goal has arisen.

A surface-level comparison of the two modes suggests that proactive control would be the preferred choice for effective self-regulation. However, this is not always true. As noted above, proactive control impinges more heavily on working memory resources because of sustained representation of goal-relevant information (e.g., making constant, conscious efforts to avoid places that might trigger cravings when trying to quit substance use). There is increased emphasis on planning to proactively ensure that the goal is met, leading to rapid mental fatigue. When events are unanticipated or when habitual responses must be withheld (e.g., in the face of temptation to use substances after substance-abuse treatment), reactive control —which is the adaptive inhibitory control mechanism—is used to regulate emotion (Brick et al., 2016). In this latter case, the coordination of cognitive systems occurs more spontaneously. Combining these perspectives, one may conclude that both proactive and reactive control are needed to maintain flexibility in the processing of information.

These two modes of inhibitory control have primarily been evaluated using the AX-CPT task (Braver et al., 2009). In this task, a cue (A or B) precedes a specific target stimulus (X or Y), and pairs of stimuli are presented (e.g., AX, AY, BX, BY). The task goal is to make a target response only to AX trials (i.e., to make a target response on X trials that follow an A cue). The AX trials are presented for a majority of the time (70% of the time), with each of the other trials (i.e., AY, BX, BY) occurring occasionally (each trial presented for 10% of the time).

In the proactive control mode, the context provided by the cue is especially helpful for correctly responding to BX trials, since the “B” cue fully predicts that the “X” probe will be a non-target. This approach is also characterized by more AY errors because participants incorrectly prepare for an “X” probe when an A-cue occurs. In the reactive processing mode, participants do not actively prepare a response during the interval between the cue and the probe. Thus, their response is dictated at the time of stimulus presentation. As a result, AY trials are easy—compared to participants who rely on proactive control—since the “Y” probe immediately implies that it is not a target response. This approach also suggests that BX trials are harder compared to participants who rely on proactive control (Cooper et al., 2017).

Within the AX-CPT testing framework, studies tend to compare the relative recruitment of these two modes. For instance, older adults show a selective age-associated decline in proactive control, but not in reactive control (Paxton et al., 2007; Bugg, 2014). Specifically, younger adults tend to utilize a proactive control approach, as reflected by increased AY errors and decreased BX errors. In contrast, older adults tend to utilize a reactive control approach, demonstrated by greater accuracy on AY trials. Furthermore, they show fewer errors on BX trials, possibly indicating successful engagement of memory recall. Collectively—relative to younger individuals—older adults’ patterns of responding are associated with a decrease in AY errors and an absence of slowing of reaction times on such trials, coupled with the disproportionate slowing on BX trials (Braver et al., 2001; Paxton et al., 2006, 2007; Rush et al., 2006; Bugg, 2014).

When conflict-interference tasks other than the AX-CPT are taken into account, the proactive-reactive control profile presents a mixed picture, with older adults showing lower proactive control as well as less efficient use of reactive control (e.g., Hasher, 2015; Xiang et al., 2016). Integrating these viewpoints, it seems plausible that proactive and reactive control need not exist at opposite ends of a continuum, although one form of control may be more efficiently deployed relative to the other (Mäki-Marttunen et al., 2019). Further, these findings point to the need for interventions in older adults that promote these mechanisms of inhibitory control.

With this aim, various performance-adaptive computerized cognitive training interventions have been proposed that purportedly target inhibitory control. Such interventions have posited that interference skills enhanced by training should result in performance gains across different contexts, not just within the specific context that an individual was trained. To date, such interventions have largely been restricted to the improvement of reactive control. Furthermore, they have not been able to show successful deployment of reactive control in contexts beyond the computer-cognitive task that was used for the training (e.g., Wilkinson and Yang, 2016, 2012; Talanow and Ettinger, 2018). The lack of efficacy of target-specific interventions signals the need for broader “engagement-based” training models. Such models are hypothesized to facilitate global enhancements in cognitive and brain functional capacity (Wilson et al., 2002; Wang et al., 2006; Park and Reuter-Lorenz, 2009). These enhancements are likely to promote efficient deployment of inhibitory control strategies. However, heretofore no interventional research has been conducted to evaluate which inhibitory control strategy is more “plastic” or amenable to change, attributable to such interventions.

One engagement-based training model that can be used to investigate this query is theater acting. Acting is a unique, multimodal, and multifactorial medium that embeds individuals in complex social contexts. Acting has been shown to impede age-related declines in cognitive domains related to problem-solving and episodic and working memory (Noice et al., 2004, Noice and Noice, 2006; Banducci et al., 2017). However, a core aspect of many acting-training models known as “active experiencing (AE)” (also known by other terms such as “staying in the moment,” “moment-to-moment-performance” and “working off your partner,” see Noice and Noice, 2018a, b for a complete discussion) involves inhibitory control. Herein, each mode of inhibitory control is embodied, cognitively and physiologically. This embodied aspect of active experiencing has differentiated it from other socially engaging interventions, such as singing and visual arts. Specifically, it has been demonstrated that active experiencing uniquely confers benefits to higher order cognitive functions relative to other art forms (Noice et al., 2004; Noice and Noice, 2008).

In active experiencing, the deliberate memorization of lines is discouraged. Instead, active experiencing is designed so that every acting instance (i.e., scene) involves the concomitant execution of proactive and reactive control. Proactive control is described as the early recruitment and monitoring of goal-directed information and is exhibited by the actor’s preemptive study of the script. Specifically, the actor attempts to break down a given scene into the smallest, ordered goal-directed actions or sub-goals. These sub-goals are called “beats.” For each beat, the actor speculates about the thoughts, attitudes, and emotions of their character, as well as what their character does – successfully or unsuccessfully – to attain a beat. To this end, the actor mentally encodes affect and motor parameters that are attuned to the beat. Thus, the actor anticipates how a situation will unfold on stage and mentally rehearses how their actor will fulfill goal-directed intentions.

At the time of script delivery—i.e., the “event”—the beat appropriate to the uttered script must be brought “online,” particularly its mental simulation. Following this event, the actor prepares delivery of the next beat. This preparation of next-beat delivery must accommodate for the participating actor’s affective response to the just-delivered beat. The participating actor’s affective response is unanticipated and constitutes the interference. That is, the participating actor’s manner of script delivery in response to the just-delivered beat might not match the actor’s mental simulation. As a result, it could interfere with the actor’s ability to sequentially access the next beat. Collectively, the preemptive study of beats and attentional monitoring of beat information to deliver the script (i.e., meet the event goals) in anticipation of interference constitutes proactive control.

After an interference has occurred, the actor must adjust their initial behavioral response (which stems from their mental simulation of the just-delivered beat) contingent to the participating actor’s non-verbal expressions. In this way, the actor’s affective expressions seek to resolve the conflict that has arisen from the actor’s internal representation of the beat and from the evolving truth of the moment, as it has arisen from the actor-actor interaction. Notably, the expression of affect and motor parameters are still constrained by the desire to meet the underlying goal (i.e., beat). Such acts of interference-resolution are characteristic of reactive control.

It is evident that an AE intervention employs both proactive and reactive control. To explore which inhibitory control strategy may be more sensitive to change and show greater benefits, a short-term AE intervention was employed in a sample of older adults. Further, we included an active control group which took a theoretical course in acting but did not actively exercise inhibitory control mechanisms during the course of the intervention. Such a group would ensure that improvements in any of the inhibitory control strategies were attributable to the engagement afforded by the AE intervention model, over and above common motivational factors associated with learning the history and background of acting.

The co-occurrence of proactive and reactive control in the AX-CPT task would make it an elegant choice to evaluate which inhibitory control strategy the AE intervention tapped more into (i.e., proactive or reactive). Indeed, the AXCPT task demonstrates interesting psychometric features in that the two trials that are the most critical to differentiating proactive and reactive control—i.e., AY and BX trials—are also the most infrequent (only 12 trials for each trial type). Notwithstanding, a recent study evaluating psychometric properties of the AX-CPT demonstrated that the reliability estimates of AY and BX trials did not increase when more trials were added (Cooper et al., 2017).

Given these observations, the AXCPT task was used to examine changes in inhibitory control across the intervention. Since these inhibitory control effects were studied within an exploratory framework, we did not make predictions on which inhibitory control strategy would show improvements compared to the other. Based on previous studies that have identified patterns of responding in older adults (Braver et al., 2001; Paxton et al., 2007; Bugg, 2014), we determined that an increase in proactive control—attributable to the engagement-based style of the intervention—would be associated with an increase in AY errors and a decrease in BX errors. In contrast, an increase in reactive control would be associated with a decrease in AY errors, no change in AY reaction time, and an increase in BX response time (Braver et al., 2001; Paxton et al., 2006, 2007; Rush et al., 2006; Bugg, 2014). Paralleling these behavioral analyses, we theorized intervention-related changes in brain activation.

Braver et al. (2009) demonstrated age-related differential recruitment of proactive and reactive control in seventeen regions of the prefrontal cortex. Given our motivation to elicit cognitive gains in older adults, we adopted a region-of-interest approach based on these regions. These regions included the middle and right inferior frontal gyrus, the inferior and superior frontal junction, and the supplementary and premotor areas. Our brain-based investigation primarily focused on the most sensitive markers of inhibitory control strategy in these regions. Thus—for each region—rather than examining the entire course of brain activation for a given trial, we focused on specific time points noted for cue-activity and probe-activity and which were related to training effects (Braver et al., 2009).

Collectively, we determined that an increase in behavioral proactive control would be accompanied by greater cue activation relative to pre-intervention and the control group in these identified brain areas. In contrast, an increase in behavioral reactive control would be accompanied by greater probe activation in these identified brain areas. We modeled both the behavioral and neuroimaging exploratory analyses using mixed models.

## Methods

### Participants

The participants were part of a larger study (*see* Banducci et al., 2017) that was approved by the Institutional Review Board at the University of Illinois at Urbana-Champaign. The participants were 179 community-dwelling adults aged 60–89 years (M = 69.46, SD = 6.59; 62% F) who on average were college-educated (*M* = 16.80 years, SD = 3.48) and provided written consent to participate in the study. They were recruited from a number of sites using a variety of sampling frames. For example, to ensure a diversity of participants, they were recruited from churches, synagogues, mosques, senior centers, and city park districts and activity centers. Additional recruitment strategies involved local media, promotional flyers, and announcements to local senior citizen agencies. Self-selection was minimized during recruitment by describing the intervention as one designed to improve cognitive health through engagement with one or more of the arts.

The participants were right-handed with an MMSE score ≥ 23 (*M* = 28.69, *SD* = 1.39; Folstein et al., 1975) and had no contraindication to MRI. Following these initial contacts, the participants were administered a battery of cognitive tests and fMRI tasks. Participants were then pseudo-randomly assigned to either the active control (*n* = 86) or the AE condition (*n* = 93); participant assignment was subject to time of enrollment. There were no age-, sex-, education-, or MMSE- related differences between the two groups. For each condition, at least 75% attendance was required for participants to be included in the study. After completing the 4-week intervention, participants returned for a post-intervention assessment (delay from the first assessment *M* = 51.48 days, *SD* = 14.78). Of the 86 participants in the control group, 4 withdrew before completing post-intervention assessment (final number of controls eligible for analysis = 82). Of the 93 participants in the AE condition, 7 withdrew before completing post-intervention assessment (final number of intervention participants eligible for analysis = 86).

### Intervention

Participants were assigned to either an active experiencing (AE)-based group that attended the intervention class, or an active control group that attended an Understanding the Art of Acting (UAA) class. Both groups met two times/week for 75-min for four weeks. The 75-min included a 15-min coffee break to facilitate additional social interaction among the group participants. Theater-actor researchers with expertise in AE interventions organized class content and trained all outside instructors. The UAA (control) class was a course in theater appreciation, featuring talks, demonstrations, and video clips of performances on stage and in film, with course topics covering the styles of acting and the history of theater. The UAA (control) condition ensured that any significant improvement in inhibitory control could not be attributed to the stimulation involved in learning about a popular and admired art form like acting.

Participants in the AE (intervention) group trained by performing short scenes with a partner (with large print scripts up to1–3 pages in length). During the preparatory phase, participants proactively and continuously investigated their character’s motivations. Following this phase, participants were encouraged to embody their character cognitively, emotionally, and physically (for a more detailed review, see Noice et al., 2015). All participants were in the same room during classes, and active feedback was provided to the acting partners.

### Behavioral Measures

The AX-CPT task was performed twice in the MRI scanner, once before randomization into groups, and once after the intervention. Stimuli were single letters presented on a black screen. The target pair was composed of AX trials, such that the appearance of an “X” probe required a target response (button press with middle finger), but only if it was preceded by an “A” cue. All other pairs – i.e., AY, BX, and BY, required non-target responses (button press with index finger). Trials were 7.5s in duration and included the following events: cue (300 m s), delay (4,900 m s), probe (300 m s), a response window (1,000 m s from probe onset), and a message window (“Trial over,” 1000 ms). There were 120 trials in total i.e., 84 AX (70%), 12 AY (10%), 12 BX (10%), and 12 BY (10%), spread across 3 scanning runs. 40 AX-CPT trials were performed in each scan.

### Neuroimaging Protocol and Pre-processing

All images were collected on a Siemens Trio 3 Tesla full body magnet, using a 12-channel birdcage head coil. Functional blood oxygenation level–dependent (BOLD) images were acquired parallel to the anterior commissure–posterior commissure (AC-PC) line with a T2-weighted echo-planar imaging sequence of 35 interleaved axial slices collected in ascending order (repetition time [TR] = 2000 ms; echo time [TE] = 25 ms; BOLD volumes = 298 for each of the 3 blocks (298x3 = 894); flip angle = 80°; field of view [FOV] = 220 × 220mm; voxel size = 3.4 × 3.4 × 4.0 mm). Structural images were acquired with a T1-weighted three-dimensional (3D) magnetization prepared rapid gradient-echo imaging (MPRAGE) protocol of 192 contiguous sagittal slices collected in an ascending manner parallel to the AC-PC line (TR = 1900 ms; TE = 2.32 ms; flip angle = 9°; FOV = 230 × 230 mm; voxel size = 0.9 × 0.9 × 0.9 mm).

#### Behavioral Analyses (AX-CPT)

In line with our behavioral exploratory analyses - we analyzed accuracy and reaction times for AY and BX cue-probe pairs. Only individuals who completed all 120 trials of the AX-CPT were included. As both accuracy and reaction times were non-normally distributed, our behavioral hypotheses were tested in generalized linear mixed models (GLMMs; see Lo and Andrews, 2015). Generalized linear mixed models are robust alternatives to transforming raw metrics (i.e., accuracy, reaction time) that conform to a standard linear regression model. Transformations often fail to address skewness problems or answer the research questions of interest, particularly in interaction-based general linear models. To circumvent this problem, generalized linear mixed-effect models (GLMMs) allow statistical analysis on the raw metric. Concomitantly, they allow meeting mathematical constraints of normalized, homoscedastic residuals as imposed by standard linear regression models (Lo and Andrews, 2015). Furthermore, unlike standard linear regression models, GLMMs are able to effectively deal with multiple sources of non-independence (e.g., participants clustered within groups, trials within a task, group assessments repeated over time; Barr et al., 2013; Brauer and Curtin, 2018).

Generalized linear mixed models are constructed from linear regression models and include three components, namely, a systematic component **X** (the linear combination of explanatory variables that form the linear predictor, as in a standard regression model), a random component **Z** (the error model which refers to the probability distribution of the response variable **y**), and a link function η (which specifies the link between the random and systematic components). Parameters are estimated using maximal likelihood (unlike standard regression models which use the least squares method), which minimizes bias of standard errors.

Presently, there is some disagreement between experts on how to construct the random component **Z**, especially when there are multiple sources of non-independence. An approach that has generally received support involves determining a “maximal random effects structure.” As a first step, this maximal random effects structure includes all random effects we might want to include depending on how the explanatory variables vary within or between levels of the variables that cause non-independence. If model convergence is not achieved at this step, the random effects structure is progressively simplified in subsequent steps until convergence is reached (Barr et al., 2013; Brauer and Curtin, 2018).

The resulting GLMM model for reaction times on AY and BX trials was as follows:

η=Xβ+Zγ

η = *g(E(**y**))* and *E*(**y**) = *h*(η), *where g*(⋅) = *link function* = ⋅ (*identity*) and *h* (⋅) = *g*^−1^(⋅) = ⋅ **y** = *h*(η) + ε, *where* **y** | γ *follows a gamma distribution*.

$${\text{X}}_{\mathrm{fixed}\mathrm{effects}}=groupID+timeID+trialID+(groupID*$$

timeID*trialID)

$${\text{Z}}_{\mathrm{random}\mathrm{effects}}=\left(1+groupID*timeID|subID\right)\left(1|trialID\right),$$

withrandominterceptsforsubIDandtrialIDandrandom

groupID*timeIDslopeforsubID

β=parameterestimatesforfixedeffects,estimatedwithγas

partofapenalizedleastsquaresstepγ=conditionalmeansor

modes,i.e.E[γ|y]

The resulting GLMM model for accuracy on AY and BX trials was as follows:

η=Xβ+Zγ

η = *g*(*E*(**y**)) and *E*(**y**) = *h*(η), $whereg\left(\cdot \right)=linkfunction=log_{e}\frac{p}{1-p}andh\left(\cdot \right)=g^{-1}\left(\cdot \right)=\frac{e^{\left(\cdot \right)}}{1e^{\left(\cdot \right)}}$

y=h(η)ε,wherey|γfollowsabinomialdistribution.

$${\text{X}}_{\mathrm{fixed}\mathrm{effects}}=groupID+timeID+\left(groupID*timeID\right)$$

$${\text{Z}}_{\mathrm{random}\mathrm{effects}}forAYtrials=\left(groupID*timeID|subID\right),$$

withrandomgroupID*timeIDslopeforsubID

$${\text{Z}}_{\mathrm{random}\mathrm{effects}}forBXtrials=\left(timeID|subID\right),withrandom$$

timeIDslopeforsubID

β=parameterestimatesforfixedeffects,estimatedwithγas

partofanon-linearoptimizationstepγ=conditionalmeans

ormodes,i.e.E[γ|y]

Analyses were completed in R studio (version 1.1.4, package “lme4,” function glmer). All analyses are presented at https://rpubs.com/saraswatiSattva/544471.

Models that were found to contribute to proactive- or reactive-control effects from the glmer analyses were evaluated for validity within a Bayesian framework. Specifically, we computed the weight of evidence for a given significant model, provided our data, compared to an alternative hypothesis involving no significant effects (i.e., a model with group × time interaction effects versus a model with only main effects, no interaction effects). This analysis was done using the brms package in R studio (function brms, version 2.14.4) with priors set using the sample_prior = “only” and pp_check function within this package. Specifically, having defined the priors, the glmer model that was found significant was validated using the brms() function, using 5 chains and 100000 iterations. Running multiple chains from different starting values and setting a high number of iterations allowed us to evaluate reliability of model convergence.

#### fMRI Analyses (AX-CPT)

We conducted all fMRI analyses using AFNI, version AFNI_2011.12.21^1^ and FSL, version 5.0.4 (Functional Magnetic Resonance Imaging of the Brain’s Software Library^2^; Jenkinson et al., 2012).

Participants’ images were preprocessed in AFNI. For each scanning run (with each run composed of 40 AXCPT trials), slice-timing correction was done with AFNI’s 3dTshift, aligning all of the slices to the first slice that was acquired by using quintic interpolation. All functional volumes were motion-corrected and realigned using cubic interpolation to the volume with the least amount of signal variability, as detected by AFNI’s 3dToutcount. The T1-weighted anatomical image was then co-registered to the same lowest-variability functional volume identified with 3dToutcount by using AFNI’s align_epi_anat.py, using a local Pearson correlation cost function (Saad et al., 2009). Next, the anatomical image was warped to MNI space, and the warps were applied to the functional volumes as well. A 128s highpass filter was applied to the time-series for each run to remove any signal drift and whiten the noise. The runs were concatenated and used as input to a general linear model (GLM).

The GLM allowed determination of parameter values for both sustained activity associated with the entire trial (state effects) and for event-related responses (effects for each stimulus). This model resulted in 8 task-related regressors (4 boxcar functions and 4 TENT functions, one per condition), and 6 movement regressors of no interest, representing the unconvolved time-series of the estimated translation and rotation in the x-, y-, and z-directions. We also included 4 drift regressors using 3dDeconvolve’s “-polort 4′ option.” The boxcar functions allowed us to independently code state effects and were 7.5 s long (equivalent to the trial length), convolved with a gamma function. The TENT functions allowed us to evaluate event-related effects for each trial type. Specifically, the TENT functions allowed us to estimate the time points within the hemodynamic response epoch—estimated as 25 s (12.5 TRs)—based on unassumed hemodynamic response shapes. Unlike the Finite Impulse Response (FIR) function which has been traditionally used in fMRI analyses, TENT has extra flexibility in that the stimuli do not have to synchronize with the TR grids. Accordingly, we were able to generate a GLM similar to Braver et al. (2009), despite differences in TR (the TR in their study was 2.5 s, while the TR in the present study was 2 s). This way, we could determine any intervention-related effects based on the general linear model used by Braver et al. to determine cue and probe- intervention effects. Thus, similar to their study, we estimated a 25-s (12.5 TR) event-related epoch for each cue-probe pair (AX, AY, BX, BY).

Notwithstanding, we accounted for differences in TR to determine hemodynamically-lagged timepoints for cue-activity and probe-activity in our study. Following a cue presentation at TR = 0 s (presented for 300 ms), the hemodynamically lagged cue-activity in Braver et al.’s study was estimated to occur at TR time points 3 (TR onset = 5 s) and 4 (TR onset = 7.5 s). These TR timepoints overlapped with our TR timepoints 3 (TR onset = 4 s) and 4 (TR onset = 6 s; see Table 1).

**TABLE 1:** TR discrepancies between Braver study and present study.

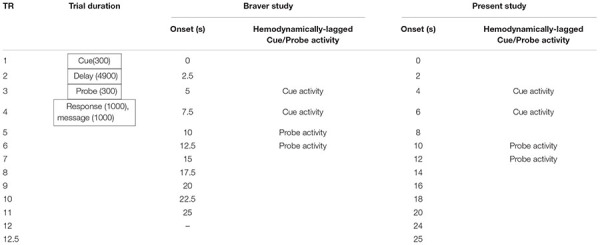

*The AX-CPT task was composed of stimuli presented as single letters presented on a black screen. Trials were 7.5s in duration and included the following events: cue (300 ms, “A” or “B” stimulus), delay (4,900 ms), probe (300 ms, “X” or “Y” stimulus), a response window (1,000 ms from probe onset), and a message window (“Trial over,” 1000 ms). This trial sequence was generally similar to the one employed by Braver et al. (2009). TRs were used as units of measurement to determine hemodynamically-lagged timepoints in response to the cue and probe stimuli. We accounted for differences in TR in Braver et al.’s study (TR = 2.5s) relative to our study (TR = 2s). Following a cue presentation at TR 1 (i.e., 0s), the hemodynamically lagged activity in response to the cue was estimated to occur at TR 3 (TR onset = 5s) and 4 (TR onset = 7.5s) in Braver et al.’s study. These TR timepoints overlapped with our TR timepoints 3 (TR onset = 4s) and 4 (TR onset = 6s). In Braver et al.’s study, following a probe presentation at TR = 5.2s, the hemodynamically-lagged probe activity was estimated to occur at TR 5 (TR onset = 10s) and 6 (TR onset = 12.5s). These TR timepoints overlapped with our TR timepoints 6 (TR onset = 10s) and 7 (TR onset = 12s). Taken together, the cue-related activity in our study was estimated to occur at TRs 3 and 4, while the probe-related activity was estimated to occur at TRs 6 and 7.*

In Braver et al.’s study, following a probe presentation at TR = 5.2 s (presented for 300 ms), the hemodynamically-lagged probe activity was estimated to occur at time points 5 (TR onset = 10 s) and 6 (TR onset = 12.5 s). These TR timepoints overlapped with our TR timepoints 6 (TR onset = 10 s) and 7 (TR onset = 12 s; see Table 1). Taken together, the cue-related activity was estimated to occur at TRs 3 and 4, while the probe-related activity was estimated to occur at TRs 6 and 7.

We identified 17 spherical regions of interest (ROIs), by creating 5 mm spheres around the peak coordinates of the 17 regions determined by Braver et al. (2009). These regions included the middle and right inferior frontal gyrus, the inferior and superior frontal junction, and the supplementary and premotor areas. Creating 5mm spheres around the peak coordinates from other studies is an established method for ROI analysis (Poldrack and Wager, 2006). We chose this approach both as an attempt to replicate the results of Braver et al., 2009, and to ensure that our ROI analysis was unbiased (Kriegeskorte et al., 2009).

We determined cue and probe-related activity for each participant at each of these 17 ROIs. Since timing is crucial to the recruitment of proactive and reactive control, we isolated each timepoint (i.e., 3, 4, 6, and 7) for examination.

Group differences at any of these time points in each of the 17 ROIs were tested in mixed models (where normality assumption was violated, non-linear mixed models were used). Generally, for each ROI at a given time point (i.e., time point 3, 4, 6, or 7), the generated mixed model was composed of fixed effects for group (intervention or control), time (pre or post), and group × time interaction, as well as random intercept effects for each participant. A family-discovery-rate (FDR) correction was applied across all cue-based models at each timepoint (i.e., separately across all models at time point 3 and separately across all models at time point 4). Similarly, an FDR correction was applied across all probe-based models at each timepoint (i.e., separately across all models at time point 6 and separately across all models at time point 7).

Finally, we were interested in identifying whether changes in performance on the AX-CPT task were associated with changes in patterns of activation on this task. To this end, we employed a linear regression framework to examine correlations between brain activity in ROIs found to be significant for intervention-related effects and behavioral indices of interest (i.e., related performance measures on AY/BX trials).

## Results

### Behavioral Results

**FIGURE 1:**
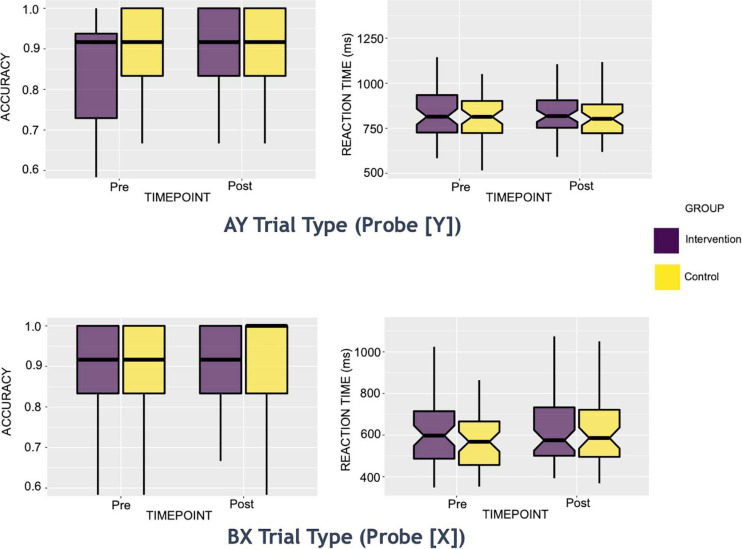
Box Plots of AY and BX trials: Behavioral Reaction Time and Accuracy Measures. These reaction time boxplots are split by group (intervention or control) for each intervention time point (pre or post). Only trials of interest to the present study are shown above (i.e., Y probe for AY trials and X probe for BX trials).

Preliminary descriptive analyses (see Figure 1 and Table 2 illustrating descriptive statistics of probe stimuli) suggested that the control group’s mean reaction times performance was least impacted on AY trials pre-to-post intervention, with a 4-ms increase in mean reaction time (standard deviation [s.d.]_*pre*_ = 132.62, s.d._*post*_ = 120.92). In contrast, the intervention group showed most improvements in mean reaction performance on this trial type, evidenced by a 7-ms faster reaction time post-intervention (s.d._*pre*_ = 180.02, s.d._*post*_ = 144.78). Both groups showed a slowing of performance on BX trials pre-to-post intervention, with the control group showing a 19-ms increase in mean reaction time (s.d._*pre*_ = 177.97, s.d._*post*_ = 162.29), and the intervention group demonstrating a 12-ms increase in mean reaction time (s.d._*pre*_ = 181.79, s.d._*post*_ = 177.62).

**TABLE 2:** Descriptive statistics for AX-CPT task.

**Trial type**	**Probe (X or Y)**	**Time**	**Group**	**N**	**Mean reaction time in ms (sd)**	**Mean accuracy (sd)**
AX	Probe (X)	Pre	Intervention	56	666.95 (121.12)	0.86 (0.19)
			Control	49	641.56 (108.45)	0.95 (0.27)
		Post	Intervention	55	665.31 (112.14)	0.94 (0.17)
			Control	50	656.57 (107.15)	0.96 (0.13)
AY	Probe (Y)	Pre	Intervention	56	852.60 (180.02)	0.82 (0.19)
			Control	49	811.24 (132.62)	0.86 (0.21)
		Post	Intervention	55	845.20 (144.78)	0.88 (0.16)
			Control	50	815.43 (120.92)	0.90 (0.13)
BX	Probe (X)	Pre	Intervention	56	617.46 (181.79)	0.83 (0.24)
			Control	49	599.66 (177.97)	0.86 (0.24)
		Post	Intervention	55	629.54 (177.62)	0.90 (0.15)
			Control	50	618.60 (162.29)	0.89 (0.20)
BY	Probe (Y)	Pre	Intervention	56	611.38 (173.53)	0.88 (0.19)
			Control	49	586.10 (129.92)	0.90 (0.21)
		Post	Intervention	55	626.82 (147.85)	0.93 (0.15)
			Control	50	599.00 (128.71)	0.95 (0.11)

*The table shows mean and standard deviation (sd) of reaction time (ms) and accuracy for each trial type, (i.e., AX, AY, BX, or BY) for each probe (i.e., X or Y). The target pair was composed of AX trials, such that the appearance of an “X” probe required a target response (button press with middle finger), but only if it was preceded by an “A” cue. All other pairs – i.e., AY, BX, and BY, required non-target responses (button press with index finger).*

Both groups showed an increase in mean accuracy pre-to-post intervention on all trial types (an increase of 1 to 5% in the control group and an increase of 5 to 8% in the intervention group across all trial types). On AY trials, the control group’s mean accuracy increased by 4% (s.d._*pre*_ = 0.21, s.d._*post*_ = 0.13), while that of the intervention group increased by 6% (s.d._*pre*_ = 0.19, s.d._*post*_ = 0.16). On BX trials, the control’s group mean accuracy increased by 3% (s.d._*pre*_ = 0.24, s.d._*post*_ = 0.20), while that of the intervention group increased by 8% (s.d._*pre*_ = 0.24, s.d._*post*_ = 0.15).

**FIGURE 2:**
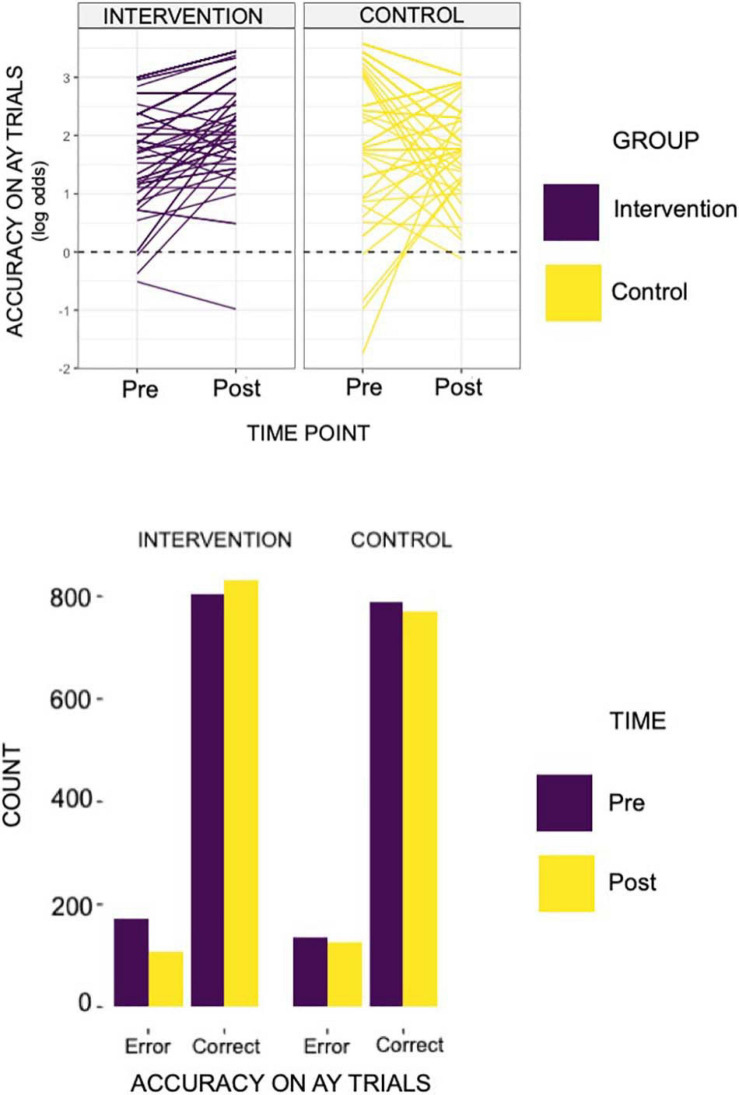
The individual line plots for each of the groups above reflects results found on the behavioral AX-CPT task. Specifically, we identified an increase in accuracy in AY trials in the intervention group, relative to controls (group × time $\hat{\beta }$ = 0.80; relative to controls and pre-intervention time point). That is to say, the acting group showed a log odds ratio of 0.80 in accuracy on AY trials relative to controls and to the pre-intervention time point. The bar plot below demonstrates the total count of error and correct trials by group (intervention or control) and by intervention time point (pre- or post- intervention).

For the planned set of analyses evaluating intervention effects of accuracy and reaction times for AY and BX, we found significant results only for accuracy on the AY trials (see Figure 2). Specifically, the intervention group showed a significant increase in accuracy post-intervention on the AY trials, relative to the control group (*α* level = 0.05; $\hat{\beta }$ for group by time interactio *n* = 0.8; *p*-Value = 0.03). We did not observe any significant intervention effects for the reaction times on the AY or BX trials.

A follow-up validation analysis within a Bayesian framework provided strong evidence for the AY-accuracy null model (i.e., only main effects, no interaction effects model). The priors that were most statistically sound for the AY accuracy model were beta-coefficients set with a normal distribution of mean 0 and a standard deviation of 2, and standard deviations set with a Cauchy distribution of mean 0 and a standard deviation of 2. With these priors, the estimated bayes factor in favor of the interaction model over the null model was 0.02. Put differently, the null model was 50 times more likely than the interaction model. Thus, we found substantial evidence that the intervention did not cause differential effects from pre-intervention to post-intervention. We also conducted a validation analysis for BX-reaction time despite no group × time differences (with similar priors). Our aim was to determine whether the interaction effects model performed better than the null model. We found evidence that the interaction effects model and the null model predicted the data equally well. Specifically, the estimated Bayes factor in favor of the interaction model over the null model was 1.00.

There was no evidence for improvement in proactive control (see Table 3 for the consolidated behavioral results and Figure 2 for a graphical representation of significant findings).

**TABLE 3:** AX-CPT behavioral results: Mixed-model framework to examine interaction effects attributed to the acting intervention.

**Analyses**	**Condition**	**Metric**	**Group effects (relative to intervention group)**$\hat{\beta_{1}}$ **(*p*-Value)**	**Time effects (relative to post-intervention)**$\hat{\beta_{2}}$ **(*p*-Value)**	**Group × Time effects (relative to intervention group, post-intervention)**$\hat{\beta_{3}}$ **(*p*-Value)**	**Bayes factor (model with interaction effects: model with main effects)**
Main	AY	ACC	−0.29 (0.30)	−0.56 (0.02)	**0.80 (0.03)**	0.02
	BX		0.13 (0.68)	−0.01 (0.96)	0.14 (0.74)	
	AY	RT	−36.86 (0.13)	19.02 (0.41)	−14.78 (0.63)	
	BX		−25.86 (0.25)	−4.34 (0.83)	5.23 (0.85)	1.00
Supplementary	AX	ACC	0.40 (0.13)	−0.24 (0.31)	−0.37 (0.27)	
	BY		−0.06 (0.91)	−0.36 (0.49)	0.50 (0.52)	
	AX	RT	−20.55 (0.02)	4.10 (0.59)	−8.08 (0.44)	
	BY		−32.59 (0.08)	−10.21 (0.54)	−3.21 (0.89)	

*Effects (expressed as a $\hat{\beta }$ coefficient) are relative to the intervention group and/or post-intervention timepoint. Bold $\hat{\beta }$ coefficients indicate significant results at *p* 0.05. Through our exploratory analysis, we found a decrease in AY errors (or an increase in AY accuracy), but no difference in BX errors, AY reaction time, or BX reaction time attributed to interventional effects. For the significant AY accuracy model, a follow-up validation analysis done within a Bayesian framework provided strong evidence in favor of the null model (bayes factor in favor of model relative to null model with no group × time effects = 0.02; that is, the null model is 1/0.02 = 50 times more likely than the interaction effects model). With respect to BX reaction time, we found that the interaction effects model and the null model both predicted the data equally well (bayes factor = 1.00). Collectively, these findings provide weak evidence for a more efficient use of reactive control.*

### fMRI Results

**FIGURE 3:**
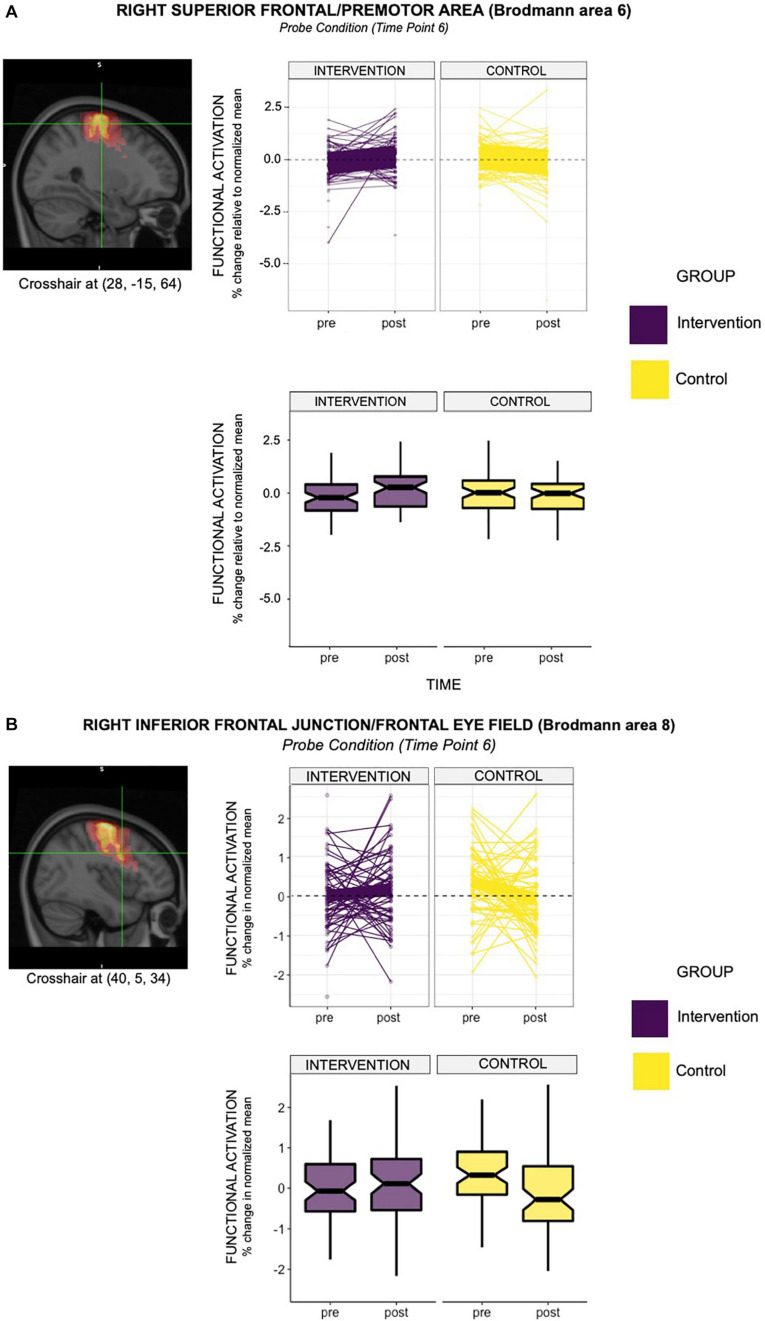
**(A)** The line graph above depicts the intervention effect found in the right hemisphere of premotor area (BA6), with the acting group showing greater functional activation in this region, relative to controls (group × time $\hat{\beta }$ = 0.61; relative to controls and pre-intervention time point). Specifically, the acting group showed a 0.61% greater activation in Brodmann Area 6 (right hemisphere) relative to controls at time point 6, when the probe was presented. The box-plot below demonstrates differences in group mean pre- and post- intervention for the two groups. It is evident from this plot that the group mean for the intervention group increased from pre- to post- intervention. This observation did not hold for the control group. **(B)** The line graph depicts the intervention effect found in the right hemisphere of the frontal eye fields (BA8), with the acting group showing greater functional activation in this region, relative to controls (group × time $\hat{\beta }$ = 0.59; relative to controls and pre-intervention time point). Specifically, the acting group showed a 0.59% greater activation in Brodmann Area 8 (right hemisphere) relative to controls at time point 6, when the probe was presented. The box-plot below demonstrates differences in group mean pre- and post- intervention for the two groups. It is evident from this plot that the group mean for the intervention group increased from pre- to post- intervention. This observation did not hold for the control group, which showed a decrease in the group mean from pre- to post- intervention.

When we examined individual timepoints, we observed that the intervention group demonstrated significantly elevated probe-activation at timepoint 6 in the right superior frontal area (Brodmann Area [BA] 6; $\hat{\beta }$ = 0.61) and right inferior frontal junction (Brodmann Area [BA] 8; $\hat{\beta }$ = 0.59) relative to controls and pre-intervention. In relation to the latter (i.e., BA8), we found some decrease in activation from pre-to-post-intervention in the control group (see box plot in Figure 3A), which may be partially driving the group × time differences observed.

Nevertheless, areas of differential activation within these observed regions (i.e., the right superior frontal region and right inferior frontal junction) predominantly overlapped with the premotor area (BA6) and frontal eye field (BA8). See Table 4 for the consolidated results, and Figures 3A,B for a graphical representation of significant findings.

**TABLE 4:** AX-CPT functional imaging (fMRI) results: Mixed-model framework to examine interaction effects attributed to the acting intervention.

**Braver’s region of interest (Talairach stereotactic space)**	**Talairach coordinates**			**MNI coordinates**			**R/L**	**Group × Time functional activation effect**$\hat{\beta }$			
								**Cue activity timepoint**		**Probe activity timepoint**	
	**X**	**Y**	**Z**	**X**	**Y**	**Z**		**3**	**4**	**6**	**7**
Middle Frontal Gyrus	24	27	50	24	24	54	R	0.03	0.02	−0.01	−0.06
	42	17	29	43	17	29		0.62	0.97	0.44	0.10
	43	28	37	44	26	38		0.24	0.31	0.13	0.11
	−35	44	32	−36	44	35	L	−0.45	−0.69	−0.26	−0.22
Right Inferior Frontal Gyrus	37	20	−6	39	24	−12	R	0.45	0.67	0.23	0.05
	51	21	0	54	24	−5		1.96	3.08	1.30	0.36
	49	39	0	51	41	−4		0.38	0.67	0.19	0.02
	−24	13	17	−25	15	17	L	0.16	0.24	0.08	0.01
	−56	13	12	−59	15	11		0.09	0.33	0.09	−0.07
	−48	27	−7	−49	31	−12		−0.77	−1.49	−0.91	−0.18
Superior Frontal	29	−10	58	28	−15	64	R	0.82	1.34	**0.61**	0.15
	−46	−7	41	−47	−9	44	L	0.84	1.42	0.63	0.15
Inferior Frontal Junction	39	6	33	40	5	34	R	0.74	1.10	**0.59**	0.16
	53	6	36	54	4	37		0.62	0.98	0.46	0.11
Supplementary and Premotor areas	15	−14	54	−4	−9	73	R	1.66	2.63	1.13	0.32
	−3	−3	65	15	−18	59	L	0.56	0.83	0.39	0.08
	−29	−12	52	−29	−15	57		0.52	0.89	0.37	0.10

*Regions of interest and corresponding Talairach coordinates were obtained from Braver et al., 2009. Each region of interest was transformed from the Talairach stereotactic space to the MNI standard space using the online conversion tool here: http://sprout022.sprout.yale.edu/mni2tal/mni2tal.html. A spherical ROI with a 5mm radius was generated from the resulting MNI coordinates using an AFNI tool called 3dUndump. R = Right hemisphere. L = Left hemisphere. The group × time functional activation effect (expressed as a $\hat{\beta }$ coefficient) corresponds to the interventional effect relative to controls and the pre-intervention timepoint. Cue-activity corresponds to timepoints 3 and 4 and is associated with the hemodynamically-lagged activity for the cue (A or B). Probe-activity corresponds to timepoints 6 and 7 and is associated with the hemodynamically-lagged activity for the probe (X or Y). Higher cue activity is associated with greater exercise of pro-active control, while higher probe-activity is associated with greater exercise of reactive control. Bold $\hat{\beta }$ coefficients indicate functional activations with family-discovery-rate (FDR) corrected *p*-Values at *q* 0.05. FDR was separately applied for cue and probe at each time point. We found significant probe-activity at timepoint 6 in the right superior frontal region (BA6) and right inferior frontal junction (BA8).*

We did not find any significant results for probe-activation at timepoint 7. No significant group × time cue-related activations were identified at any of the timepoints. Collectively, these results suggest an increased recruitment of reactive control with temporal specificity, attributed to engagement in the intervention.

Based on these previous findings, we examined the relationship between behavioral indices of interest (AY accuracy, BX reaction time) and significant brain activity (i.e., BA6 and BA8 at timepoint 6). We did not find a significant relationship between the observed increase in AY accuracy and the observed increase in BA6 and BA8 probe-activity attributed to the intervention (Table 5). Although we found a significant relationship between change in BX reaction time and change in probe-activation, this latter result is not interpretable given the lack of significant behavioral findings (i.e., there were no group × time effects in BX reaction time; Table 5). Collectively, we did not find a meaningful relationship between behavioral and brain activity.

**TABLE 5:** Brain-behavior interactions based on significant findings.

**Behavioral index *Δ = Post-Pre***	**Brain ROI**	**Probe time pt.**	**Intervention group × Δ behavioral index Beta-coefficient (p-value)**	**Description**
Δ AY accuracy	Right Superior Frontal (BA6)	6	0.02 (0.98)	*Relative to the control group and controlling for pre-brain activation in BA6 at timepoint 6*, a 1 unit increase in AY accuracy from pre to post in the intervention group was associated with a 0.02 unit increase in post-activation at BA6 at this timepoint. However, this association was not significant.
	Right Inferior Frontal Junction (BA8)	6	0.02 (0.99)	*Relative to the control group and controlling for pre-brain activation in BA8 at timepoint 6*, a 1 unit increase in AY accuracy from pre to post in the intervention group was associated with a 0.02 unit increase in post-activation at BA8 at this timepoint. However, this association was not significant.
Δ BX reaction time	Right Superior Frontal (BA6)	6	0.0042 (0.01)	*Relative to the control group and controlling for pre-brain activation in BA6 at timepoint 6*, a 1 unit increase in BX reaction time from pre to post in the intervention group was associated with a negligible increase in post-activation at BA6 at this timepoint. This association was significant.
	Right Inferior Frontal Junction (BA8)	6	-0.0007 (0.69)	*Relative to the control group and controlling for pre-brain activation in BA8 at timepoint 6*, a 1 unit increase in BX reaction time from pre to post in the intervention group was associated with a negligible decrease in post-activation at BA8 at this timepoint. However, this association was not significant.

*We employed a regression framework to examine partial correlations between intrinsic brain activity (in BA6 and BA8 at timepoint6) and intervention-related behavioral indices of interest (i.e., AY accuracy and BX reaction time). Specifically, we modeled the relationship between post-intervention activity at BA6 with post-minus pre-change in AY accuracy, baseline pre-intervention activity at BA6 and group as covariates. For example, the regression equation for Δ (change in) AY accuracy at BA6 was: *BA6 post-intervention activity at timepoint 6 ∼ groupID + BA6 pre-intervention activity at time point 6 + Δ AY accuracy*. Thus, each beta-coefficient representing an interaction effect is referenced to the control group, controlling for pre-intervention activity at BA6 at timepoint 6. We constructed similar models for BA8 as well as BX reaction time (at BA6, BA8, timepoint 6). With respect to *Δ* AY accuracy, none of the interaction effects were significant. That is, the observed behavioral increase in AY accuracy in the intervention group did not correspond with observed brain changes in BA6 and BA8. With respect to *Δ* BX reaction time, we found a significant association between intervention-related change in BX reaction time and BA6 activity at timepoint 6. However, these associations are not interpretable since we did not find significant behavioral differences in BX reaction time across groups and over time (see Table 3). All models satisfied assumptions of normality and homogeneity of variance. The presence of significant effects was evaluated at the level of alpha < 0.05.*

## Discussion

Older adults tend to show declines in both proactive and reactive modes of inhibitory control. Therefore, there is a need for interventions that can promote inhibitory control in this population. Engagement-based interventions are theorized to enhance global cognitive and brain plasticity, with implications for gains across multiple cognitive domains, including inhibitory control. However, heretofore no research has been conducted to evaluate which mode of inhibitory control (i.e., proactive or reactive) may be more “plastic,” attributable to such interventions.

To investigate this query, a 4-week active experiencing (AE) intervention was employed in a sample of older adults using a randomized control design. The acting intervention required the active deployment of proactive and reactive control mechanisms. The active control group took a theoretical course about acting. The AX-CPT task was used to examine changes in inhibitory control across the intervention. Since inhibitory control-intervention effects were examined within an exploratory framework, we did not make predictions on which inhibitory control strategy would show improvements compared to the other. We note that our study had substantially more participants than previous studies that employed the AX-CPT task, lending credence to the behavioral and neuroimaging findings on this task.

On the behavioral AXCPT, the intervention group showed an increase in AY accuracy without any behavioral costs (since BX reaction times did not show intervention effects), possibly indicative of efficient use of reactive control. However, we found strong evidence supporting the no-interaction effect model, relative to the demonstrated model showing an increase in AY accuracy in the intervention group.

From a neuroimaging standpoint, we found robust evidence for intervention-related effects in brain areas that survived FDR correction at timepoint 6. Specifically, the intervention group showed functionally greater probe activation (timepoint 6) in the right superior frontal and inferior frontal junction areas, corresponding to BA6 and BA8 respectively. Both BA6 and BA8 have been directly implicated in reactive control processes (Brass et al., 2005; Forstmann et al., 2008; Braver et al., 2009). Both areas have also been associated with orchestrating complex visuomotor interactions (Mastropasqua et al., 2020), which are characteristic of the reactive control aspect of active experiencing. Integrating these viewpoints together, it is likely that BA6 and BA8 facilitate deployment of reactive control.

Our study found a preference for probe-related regional activation in the right hemisphere. The right hemisphere —in right handers, as in the present study— specializes in vestibular processing, which allows an individual to orient themselves in space based on visual and proprioceptive cues (e.g., body posture and orientation; Mast et al., 2014; Besnard et al., 2015; Dieterich and Brandt, 2015). Within this context, the right frontal eye field (implicated in the present study) has been uniquely identified as important to visuospatial perception (Mastropasqua et al., 2020). Collectively, the preference for right-hemisphere activation in these brain areas suggest an increased emphasis on regulating visuomotor expressions, likely facilitated by repeated deployment of reactive control mechanisms in response to spontaneously evolving situations on stage. These observations bolster our interpretation that there were group × time differences of activation in brain areas associated with reactive control.

It is worth emphasizing that there was a temporal specificity in the intervention-related activation in BA6 and BA8, which occurred only at TR timepoint 6. This result may represent the peak hemodynamic response. Specifically, given the presentation of the probe stimulus (“X” or “Y”) from 5.2s to 5.5s (see Table 1), the hemodynamically-lagged activity would be expected to occur 4-6s thereafter (i.e., 9.5s to 11.5s). Our finding suggests that the probe-related activity occurred during an early part of this window, i.e., at 10s (or the interpolated snapshot at TR 6).

Finally, we note the disparity in our behavioral and brain results, as illustrated by the lack of significant brain-behavior relations in AY accuracy (Table 5). We estimate that there were neural effects related to reactive control that did not manifest behaviorally. From this perspective, brain changes may be a more sensitive measure of training related effects-especially given the short time span within which such effects were evaluated (4 weeks between pre- and post-testing).

We acknowledge that our engagement-based acting intervention cannot be operationalized as a cognitive intervention given our study findings as well as the observation that our control group was not cognitively taxed, making comparisons on cognitive gains difficult to infer. Instead, from our study results, we are able to postulate that engagement in acting may promote certain aspects of reactive control relative to a control group that simply learned about acting. We suggest that to effectively evaluate benefits associated with broad-based engagement training models, it would be helpful to generate study designs that compare such models to cognitive interventions that emphasize adaptive learning (e.g., Finc et al., 2020).

Further, we recognize that there were no benefits to proactive control, suggesting that acting interventions—and by extension, other broad-based intervention models that are not targeted at specific cognitive skills— may not be well-suited for the promotion of proactive strategies in the aging population and/or must be implemented over a longer time course.

## Conclusion

We conducted a 4-week broad-based acting intervention in older adults, designed as a form of cognitive engagement. We explored which mode of inhibitory control (proactive or reactive) would be more amenable to change and show greater benefits attributable to engagement with the intervention. The intervention group participated in active experiencing, an acting model that involves active deployment of proactive and reactive control mechanisms. The control group learned about the history and styles of acting. We found weak evidence for improvements in reactive control, attributed to engagement versus learning about acting. Notwithstanding, we found brain-related benefits in reactive control. We did not find any evidence for improvements in proactive control. Future studies may benefit from broad-based interventions that employ cognitively-taxed control groups, more assessment time points, and which tap into concrete as well as more dimensional aspects of cognitive functioning. Such designs would be able to better illustrate specific mechanisms of change attributed to such interventions.

## Data Availability Statement

The raw data supporting the conclusions of this article will be made available by the authors, without undue reservation.

## Ethics Statement

The studies involving human participants were reviewed and approved by the Institutional Review Board-University of Illinois at Urbana-Champaign. The patients/participants provided their written informed consent to participate in this study.

## Author Contributions

TN, HN, and AK designed and directed the project. AR and AJ developed the conceptual framework and performed all analyses. AD aided in interpreting the results. All authors provided critical feedback and helped shape the research, analysis, and manuscript.

## Conflict of Interest

The authors declare that the research was conducted in the absence of any commercial or financial relationships that could be construed as a potential conflict of interest.

## Footnotes

1 https://afni.nimh.nih.gov/

2 https://www.fmrib.ox.ac.uk/fsl
